# Fast electro-plasmonic detection of heart signal in Balb/C cells onto one-dimensional plasmonic grating

**DOI:** 10.1371/journal.pone.0282863

**Published:** 2023-03-16

**Authors:** S. Akbari, S. M. Hamidi, H. Eftekhari, A. Heirani-Tabasi

**Affiliations:** 1 Magneto-plasmonic Lab, Laser and Plasma Research Institute, Shahid Beheshti University, Tehran, Iran; 2 Plasma Physics Research Center, Science and Research Branch, Islamic Azad University, Tehran, Iran; 3 Research Center for Advanced Technologies in Cardiovascular Medicine, Tehran Heart Center Hospital, Tehran University of Medical Sciences, Tehran, Iran; Universiti Brunei Darussalam, BRUNEI DARUSSALAM

## Abstract

The heart is a vital and complex organ in the human body that forms with most organs between the second week of pregnancy, and fetal heart rate is an important indicator or biological index to know the condition of fetal well-being. In general, long-term measurement of fetal heart rate is the most widely used method of providing information about fetal health. In addition to fetal life, growth, and maturity, information such as congenital heart disease, often due to structural or functional defects in heart structure that often occur during the first trimester of pregnancy during fetal development, can be detected by continuous monitoring of fetal heart rate. The gold standard for monitoring the fetus’s health is the use of non-invasive methods and portable devices so that while maintaining the health of the mother and fetus, it provides the possibility of continuous monitoring, especially for mothers who have a high-risk pregnancy. Therefore, the present study aimed to propose a low-cost, compact, and portable device for recording the heart rate of 18-day-old fetal mouse heart cells. Introduced device allows non-invasive heart rate monitoring instantly and without side effects for mouse fetal heart cells. One-dimensional gold-plated plasmonic specimens as a physiological signal recorder are mainly chips with nanoarray of resonant nanowire patterns perform in an integrated platform. Here the surface plasmon waves generated in a one-dimensional plasmonic sample are paired with an electrical wave from the heart pulse, and this two-wave pairing is used to record and detect the heart rate of fetal heart cells with high accuracy and good sensitivity. This measurement was performed in normal mode and two different stimulation modes. Stimulation of cells was performed once using adrenaline and again with electrical stimulation. Our results show that our sensor is sensitive enough to detect heart rate in both standard and excitatory states and is also well able to detect and distinguish between changes in heart rate caused by different excitatory conditions.

## 1. Introduction

The heart and circulatory system are the first organs to develop in the fetus, and the fetus experiences its first heartbeat in the third week of life [1]. Anatomically, the physiological function of the fetal heart and the neonatal heart have different mechanisms. Also, during pregnancy, fetal heart circulation is different in the comparison with neonatal circulation. Briefly, the heart’s job during the fetus is to pump oxygenated blood throughout the body, including the lungs [2]. Fetal heart rate (FHR) is a critical parameter that can be monitored and indicators to ensure fetal health [3–5]. When the heart beats, it pumps oxygenated blood throughout the fetus. Getting enough oxygen to the fetus is crucial to preventing hypoxia, which affects the whole body of the fetus. Hypoxia is a very dangerous case, and fetal cerebral blood flow may be significantly reduced. Observing any changes in the normal rhythm and pattern of fetal heart rate, such as decelerations, loss of high-frequency variability, and pseudo-sinusoidal may indicate fetal asphyxia [6, 7]. In the case of prolonged and severe hypoxia, the fetus can suffer nerve and brain damage, and even if the fetus suffocates severely enough, it can lead to fetal death [8]. As a result, continuous monitoring of fetal heart rate enables rapid and early detection [9, 10].

Therefore, over the years, studying fetal heart rate has attracted scientists’ attention to finding a safe and sure method for monitoring fetal heart rate. Also, in recent decades, remote health monitoring, based on non-invasive, wireless [11] and also wearable sensors [12, 13], has provided an efficient and cost-effective solution to home monitoring aims. Patients can easily monitor their vital signs at home, such as heart rate, blood sugar, blood pressure, blood oxygen, and ensure their health. For this purpose, we tried to develop a light and portable plasmonic biosensor for real-time monitoring of the heart rate of mouse embryos.

Optical Surface plasmon resonance (SPR) biosensors represent the most advanced and developed optical label-free biosensors. These biosensors are a powerful detection and analysis tool that has vast applications in environmental protection, biotechnology, medical diagnostics, drug screening, food safety, security [14, 15] or pressure sensors based on some simple or complicated nanostructures like as Metal-Insulator-Metal waveguide [16] or Intersection Nanostructure [17].

In our earlier work, we introduced a flexible, miniaturized and wearable plasmonic sensor that could record the frog’s heartbeat instantly with acceptable accuracy and sensitivity [18]. That report, was introduced based on surface lattice resonance (SLR) in two-dimensional plasmonic substrates to enhance the sensitivity and get a fast response. Due to this fact that in that two dimensional structures, we have resonance based on coupling of nanorods and diffraction orders in two dimensions, we want to examine shorter unit cells in one dimensional plasmonic gratings as a main substrate and also instead of living animals, use of cells to get earlier diagnostics. In this study, we introduced a small, portable sensor to record the instantaneous heart rate signal of mouse fetal heart cells cultured on a one-dimensional plasmonic substrate.

The main mechanism of our sensor’s operation is based on the resonance of surface plasmons in such a way that when the red laser shines on the interface between dielectric and metal, which is gold on the plasmonic substrate, surface plasmon resonance occurs and the SPP wave will be generated. Then this SPP wave is paired with the electrical wave of the heart and actually, a modulation occurs. Here the modulation is the amplitude modulation and the amplitude of our SPP wave is modulated. If the phase modulation occurred because the phase has a higher sensitivity, A series of additional sharp peaks would have been created than what we have here [19].

## 2. Material and methods

### 2.1. Plasmonic substrate and microchip

The main challenge of this study was the viability of cardiac cells cultured on a one-dimensional plasmonic substrate. As long as the cells are alive, they must be kept under special conditions in a sterile incubator environment and all tests must be recorded inside the incubator. Because if the cells are removed from the incubator environment, due to the change in temperature, the heart rate of the cultured heart cells is greatly reduced, and after one hour due to non-sterile laboratory environment and the lack of survival conditions for the cell, they lose their lives and their pulse is cut off. Therefore, it was necessary to design a small, portable sensor to be easily sterilized and placed in a small incubator space like as the microchip in the DVD-ROM building in our earlier work [20].

**Fig 1:**
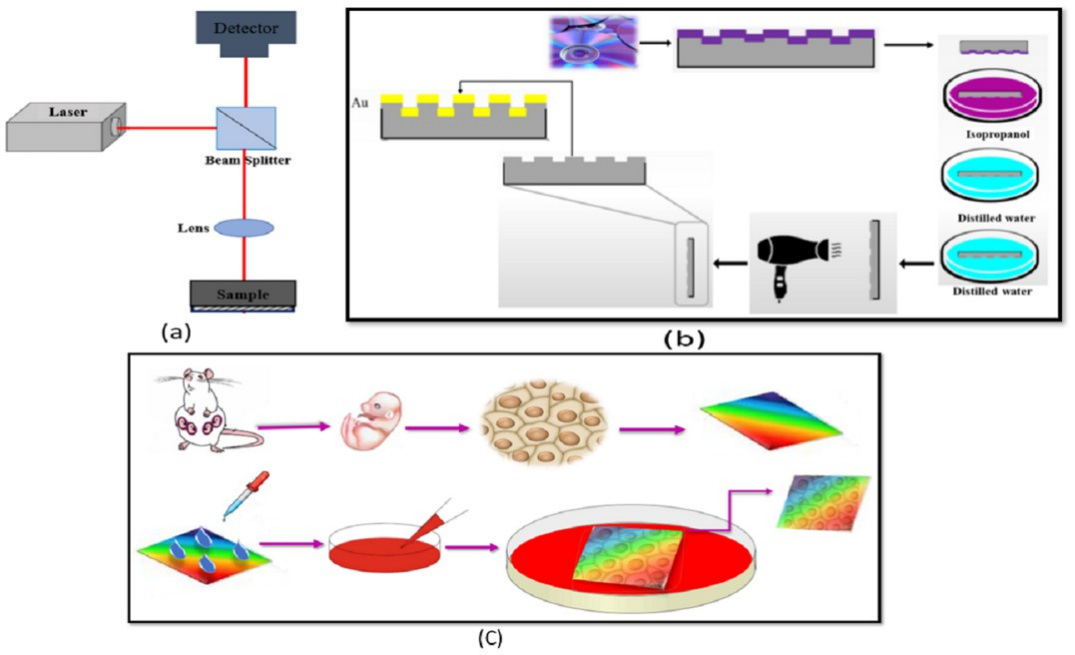
(a). Optical setup of microchip and (b) Schematic of the fabrication process flow for 1D plasmonic substrate and (c) Schematic of mouse fetal heart cell extraction and culture process on one-dimensional plasmonic substrate.

According to Fig 1(a), the internal structure of the microchip consists of a laser, a lens to focus the laser light, a Beam splitter, and a photodetector to collect the reflected light from the surface of the sample. However, changes must be made to the structure of the microchip so that it can be used as a sensor to record high-sensitivity heart signals.

The first change was done in a type of the laser because the laser wavelength in the main microchip structure was 780 nm and was not suitable for sensing work. Therefore, the laser in the main microchip structure was removed and a semiconductor laser diode, 680 nm, with 5 mW power was replaced. Another change that has taken place was the change of the main set of photodetector to one high quality single channel ones. After applying these changes, the microchip was fully prepared and could be used as a small, lightweight, portable, high-sensitivity sensor to record the heartbeat instantly.

To explain the process of making a one-dimensional plasmonic sample, a raw CD was cut with scissors and cut in half in the middle, then the two layers of CD that were glued together were separated and the outer shell was discarded, and only the inner layer was held and then the layer was cut into small pieces with scissors. Next, the purple color that is placed on the CD should be removed and colorless CD parts should be produced. For this purpose, each of the created parts should be placed in isopropanol for 5 minutes, and after that, when the purple color was thoroughly cleaned, washed twice with distilled water to thoroughly clean and remove all stains, and finally, immediately after the second rinse with distilled water, the desired part was dried with a dryer so that the surface of the part was arid and no drops left on it. Now that the colorless pieces have been obtained with a one-dimensional periodic pattern, they are gently glued to a slide so that no fingerprints or contaminants are formed on them, and in the final step, a thin layer of gold 35 nm thick is coated to the parts by DC sputtering method. We have a one-dimensional plasmonic sample with a periodic pattern, 780 nm, and a thin layer of gold (Fig 1b). The advantages of the produced structure include a fast and cheap manufacturing process, small dimensions, and good strength.

Another sample produced is a one-dimensional plasmonic substrate that is made according to the method mentioned above, and the only difference between this type of sample and the sample described in the previous section is the connection of two electrodes to the sample surface. To create electroplasmonic properties in the plasmonic sample, a voltage in the specified range of 2 to 5 volts must be applied to the sample’s surface. Due to the coating of a thin layer of gold on the plasmonic sample, the two electrodes must be connected to the substrate with conductive silver adhesives.

### 2.2. Cell Culturing

One-dimensional plasmonic substrates were thoroughly sterilized before cell culture began. Conventional plasmonic substrates were sterilized for one hour under UV light, and electrode-bound plasmonic substrates were sterilized with plasma gas at 60° C for 25 minutes.

After sterilization of the substrates, the surface of the substrates must be pre-covered with a layer of human collagen. Therefore, 1 mg per ml of human collagen was poured onto the substrates and incubated for 30 minutes at room temperature. In samples with no collagen coating on the substrate, no connection was made between heart cells and plasmonic substrates and the cells did not adhere to the substrate surface.

Cardiomyocytes were extracted from the embryos of 18-day-old mice. Briefly, the fetal hearts of three 18-day-old pregnant mice were isolated and completely crushed in Dulbecco phosphate buffer (DPBS) without calcium and magnesium (Gibco, Grand Island, NY) and pen/strep 1X. Cells were digested with 1% type II collagenase (Invitrogen) and cultured in DMEM F12 medium containing 10% FBS, 1X penicillin-streptomycin (Gibco-BRL), and 10 nM bFGF at 37° C in 5% CO_2_ [21].

Finally, plasmonic substrate covered by collagen was placed in a Petri dish containing culture medium and submerged cells in it were placed in an incubator for one week at 37°C and 5% CO_2_ gas as shown in Fig 1(c). After a week, the adhered cells to the substrate had pluripotent heartbeat so that their heart beats could be easily seen under a microscope.

### 2.3. Recording signal process

**Fig 2:**
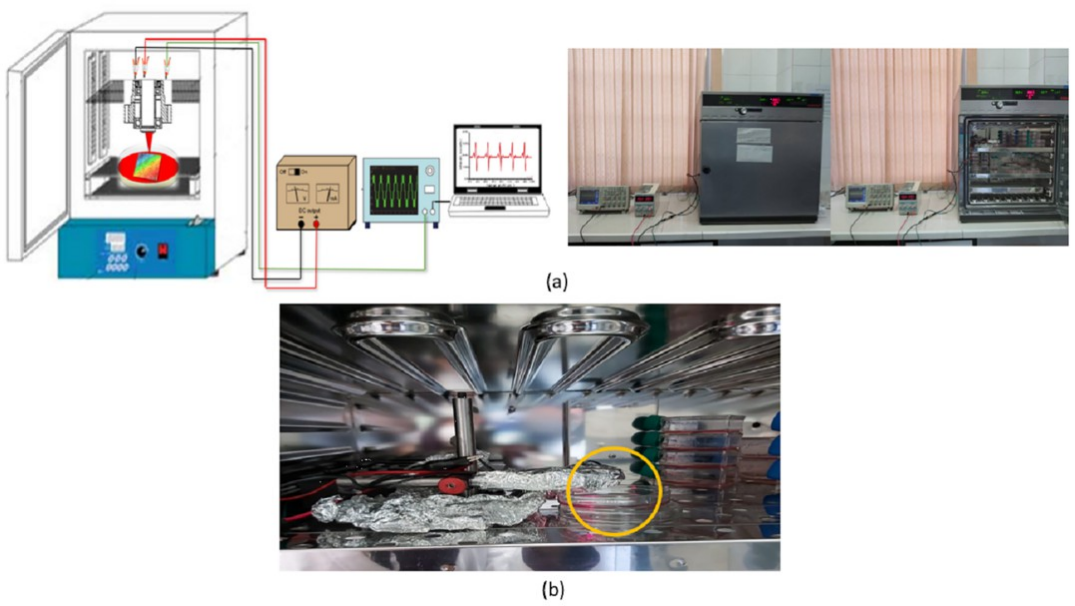
(a). Main experimental setup in the incubator for heart signal recording and in the inset: real pic of experimental setup and (b) the microchip at a suitable distance from the sample.

The microchip and its support base were thoroughly disinfected with alcohol, and then the microchip, its support base, and the wires were coated with aluminum foil to prevent any contamination from entering the incubator environment. The microchip was then placed inside the incubator and the wires connected to the power supply and oscilloscope were removed from the incubator (Fig 2a).

According to Fig 2b, the microchip was placed at a suitable distance from the sample and after adjusting the distance, the incubator door was closed immediately so that the cells were in perfect condition. At first, the cells were in a completely normal situation and there was no additional factor that could alter the heart’s normal signal. Therefore, the heart signal of heart cells was recorded in a completely normal state. Next, adrenaline and diluted adrenaline were added to the culture medium in the Petri dish in which the substrate and the cultured cells were immersed. The adrenaline was a strong stimulant for the cells and caused changes in the normal heart rhythm of cardiomyocyte cells. The last step of the test was to record the heart signal of the cultured cells on a one-dimensional plasmonic substrate with electrodes attached to both ends. The electrodes installed at both ends of the sample were applied with a voltage supply in the specified range of 2 to 5 volts, and at the same time as the voltage was applied to the surface of the plasmonic substrate and the cells cultured on it, the heart signal of the cells was recorded. In fact, the heart signal was recorded at electrical stimulation of cells (electric shock to the cell).

**Fig 3:**
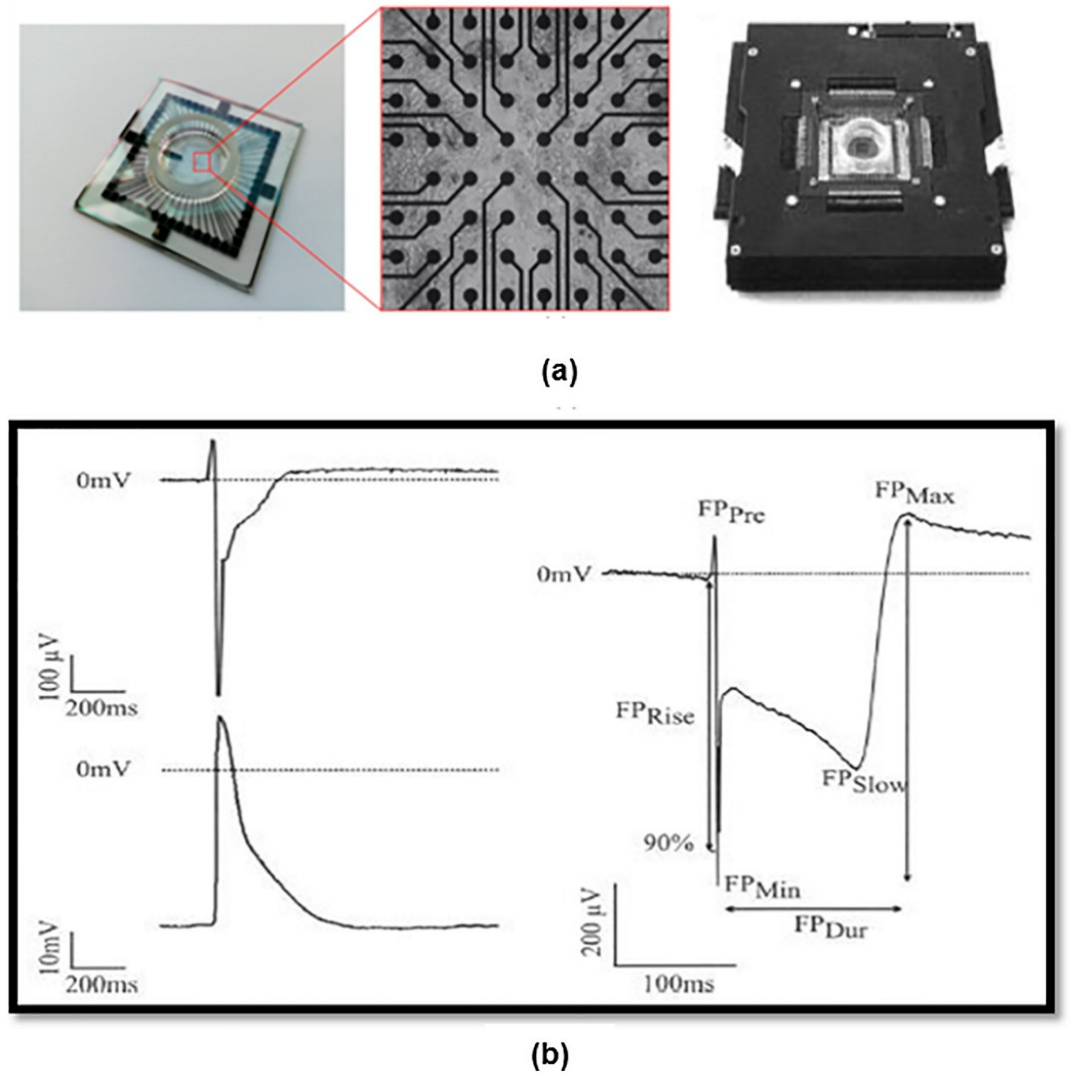
(a) Picture of a multi-electrode array plate, amplifier, and electro gram. (b) An electro gram is matched and compared with the action potential.

In addition, to get more sense of cells, we get Scanning electron microscopy (SEM), optical microscopy and also MTT analysis and we record the heart signal of mouse fetal heart cells was also recorded using the electrophysiological method according to [22]. Previously, the recordings were usually in the form of membrane potential recordings and currents through the single-cell membrane using the patch-clamp technique, but with the advent of human cardiac micro-tissue production technology using pluripotent stem cells (hips) differentiation, this type of recording which is very similar to electrical recordings from the whole heart or electrocardiogram has become common and has provided useful information about the depolarization and repolarization phases as well as the length of a contractile cycle. To record this, plates containing a multi-electrode array are used, which are connected to an amplifier and output the amplified electrical signal in the form of electrical waves (Fig 3).

In the electrophysiology method, the extracellular potential is recorded and, in this picture, an electrogram of the cells’ function which contains important information in the field of repolarization and depolarization phases, as well as a contraction cycle of the cells, is matched and compared with action potential (Fig 3).

### 2.4. Human sample statement

This study used collagen as an adherent substance to which cells were attached. Collagen was commercially provided (human collagen type I, Sigma-Aldrich) and was not extracted from human tissue.

### 2.5. Sample guideline

All experimental protocols in this study was approved by the Ethics Committee of the "Ethical committee of Vice president of research of Shahid Beheshti university/ IR.SBU.REC.1400.219.

### 2.6. Statement

All methods were carried out in accordance with relevant guidelines and regulations.

## 3. Results and discussion

**Fig 4:**
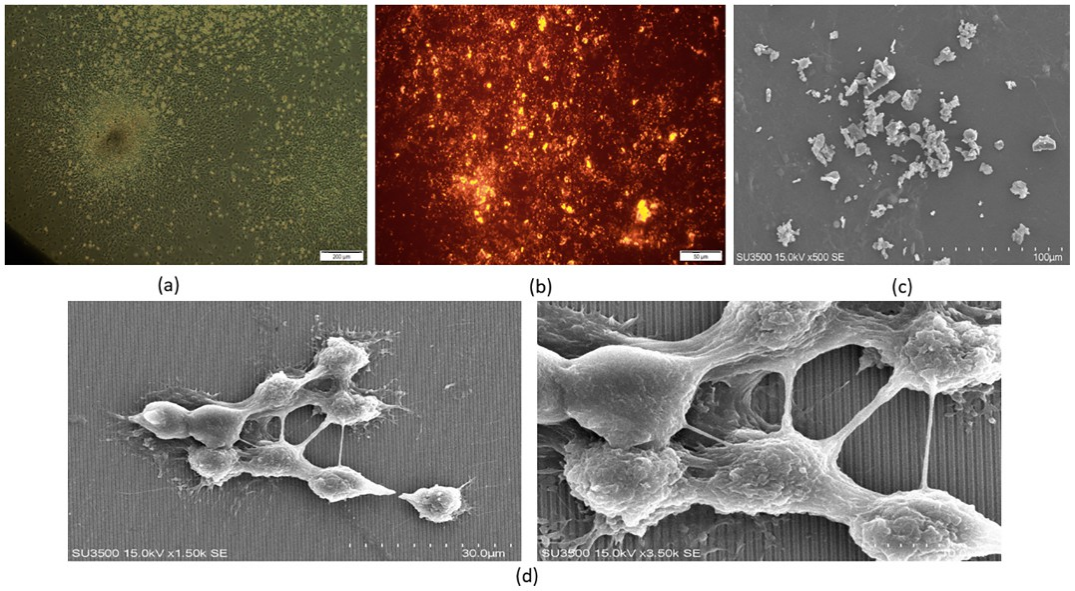
(a). Normal image of cardiomyocyte cells under an inverted microscope, (b). Image of cardiomyocyte cells stained with a DiI dye under an inverted microscope, (c) SEM image of cardiomyocyte cells cultured on cell culture plate, (d) SEM image of cardiomyocyte cells cultured on a one-dimensional plasmonic substrate.

Cells implanted on a one-dimensional plasmonic substrate were imaged under an inverted microscope (Olympus’ IX53 inverted microscope) in two modes. Fig 4a cells were usually placed under a microscope. Fig 4b the cells were first stained with DiI color and then placed under a microscope. For this purpose, one microliter of DiI dye was diluted in 500 μl of PBS and added to the microtube containing 1 million cells in suspension. The samples were then incubated for 30 minutes at 37° C and dark. The cells were then washed twice with PBS by centrifugation at 400 g. Finally, the stained cells were cultured on a one-dimensional plasmonic substrate. Fig 4c shows the SEM image of cells cultured on a cell culture plate, and Fig 4d shows the SEM image of cells cultured on one-dimensional plasmonic substrate.

**Fig 5:**
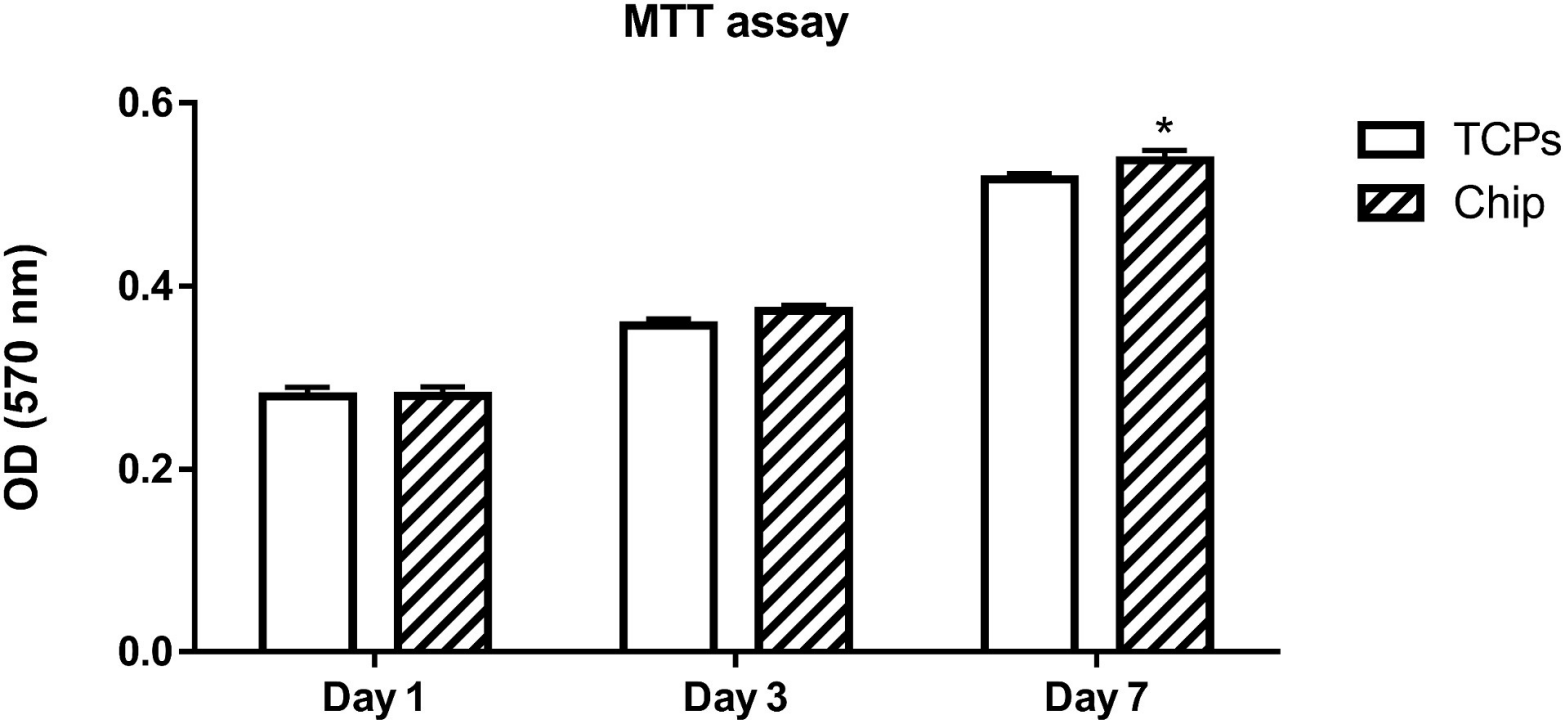
MTT test results for both groups of cells, cells cultured on a tissue culture plate (TCPs) and cells cultured on a one-dimensional plasmonic substrate (Chip).

MTT test was performed for both groups of cells which implanted on a tissue culture plate and on a one-dimensional plasmonic substrate as shown in Fig 5. The results of the MTT test had taken from a one-dimensional plasmonic substrate showed that our chip had no toxic properties and the cells survived on it. On the first and third day of the MTT test results of cell culture on both substrates, the tissue culture plate (TCP) and the one-dimensional plasmonic substrate (Chip) were similar, but on the seventh day, there was a significant increase for the chip substrate, which can be interpreted as a result of the presence of collagen layer on the plasmonic substrate. The collagen layer Created adhering properties so that more cells could be stuck to a chip.

**Fig 6:**
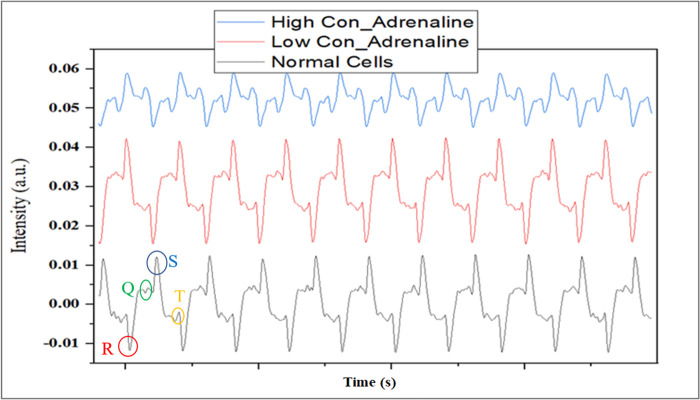
Heart signal from cardio myocyte cells, main signal (first row from bottom), and the signal recorded in stimulated positions with diluted and pure adrenaline.

After all of these confirmations onto cell fixation and live cells, heart signals from the cells were recorded in three different situations as normal (First row from bottom) and excited by adrenaline (second and third rows from bottom). As shown in Fig 6, after adding diluted adrenaline to the cell, changes in the Q and T domains are pretty evident, but no changes are made in the R and S domains. After adding pure adrenaline to the cells, all four amplitudes of the heart wave have undergone fundamental changes, so that the amplitude Q appears as a deep and the amplitude T as a peak, and the amplitude of the S and R waves has changed significantly.

In the second step, to get confirmation of electroplasmonic sensitivity, a plasmonic substrate with a resistance of about 65 ohms was applied with a voltage of 4.7 volts and currents ranging from 0.03 amps to 0.06 amps. By applying different voltages and currents, the cultured cells on the plasmonic substrate experienced an electric shock and as a result, their heart signal went out of normal. It is obvious that this stimulator can use to control the normal heart signals which come from any other disease in inverse side.

**Fig 7:**
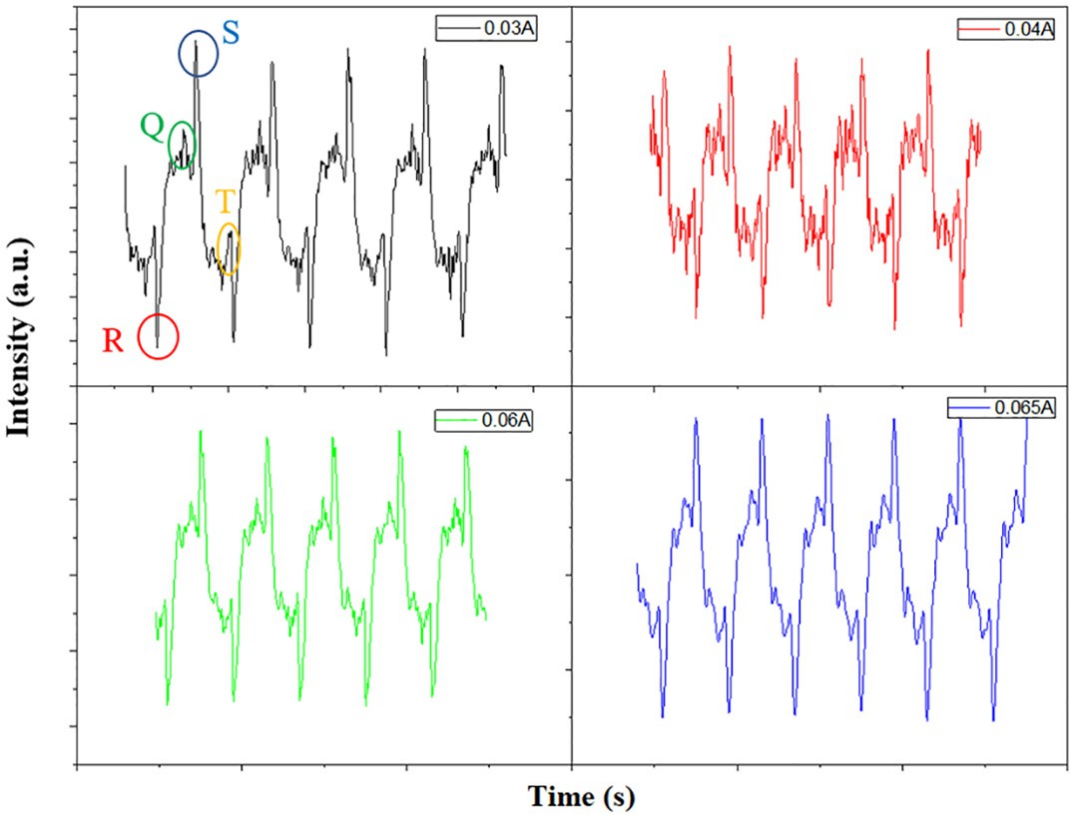
The recorded heart signal from the cardiomyocyte cells in the stimulated state due to the applying different electric currents.

As shown in Fig 7, due to the applying different currents, the heart rate experiences an arrhythmia, which means that changes in heart rhythm are different and do not follow a specific pattern.

In this electroplasmonic substrates, by ignoring the capacitive effects, a potential applied across the surface of the plasmonic substrate (V_0_) creates a surface charge density at the gold surface [22–24]. The shift in the resonance wavelength as a result of an applied voltage V_0_ can be written as:

$$\Delta \lambda_{\text{LSP}}=-[\varepsilon_{0}\omega_{\text{P}}{}^{*2}\lambda_{\text{LSP}}{}^{3}/8\pi^{2}{\text{c}}^{2}\text{N}\;\text{e}\;{\text{d}}_{\text{TF}}(\varepsilon_{\infty }+1-\text{L}/\text{L})]{\text{V}}_{0},$$

where λ_LSP_ is the localized surface plasmon (LSP) resonance wavelength, ε_0_ is the electric permittivity of vacuum, ω_P_ is gold plasma frequency, d_TF_ is Thomas-Fermi screening length and L is the geometrical factor in the polarization direction of the incident electromagnetic wave and N = 5.9×10^22^ cm^-3^.

Now, by placing the amount of each of the parameters in the above equation, it can be argued that the LSP resonance change is so tiny that the deformation of the transmission spectrum can be ignored [25] and it can be assumed that Δλ = Δλ_LSP_. Therefore, it can be concluded that applying voltage to the surface of a one-dimensional plasmonic substrate does not change the resonant wavelength of surface plasmons.

Briefly, when voltage is applied to the plasmonic substrate, there is no change in the resonant wavelength of the surface plasmons, so there is no change in the property of the plasmonic substrate. However, the one-dimensional plasmonic substrate to which the electrode is attached and cultured cells on the substrate can be considered similar to an electrical circuit with two parallel resistors. When a certain voltage is applied to the circuit, it splits between two resistors (one plasmonic substrate and the heart cells fixed on it) and most of it passes through the smaller resistor. So we used a 67-ohm plasmonic substrate, which was a relatively higher resistance than the heart cells. As a result, when current flowed through the substrate, a more significant portion of it passed through the cells, which was an electric shock to the cells and caused an arrhythmia in the normal heart rate.

**Fig 8:**
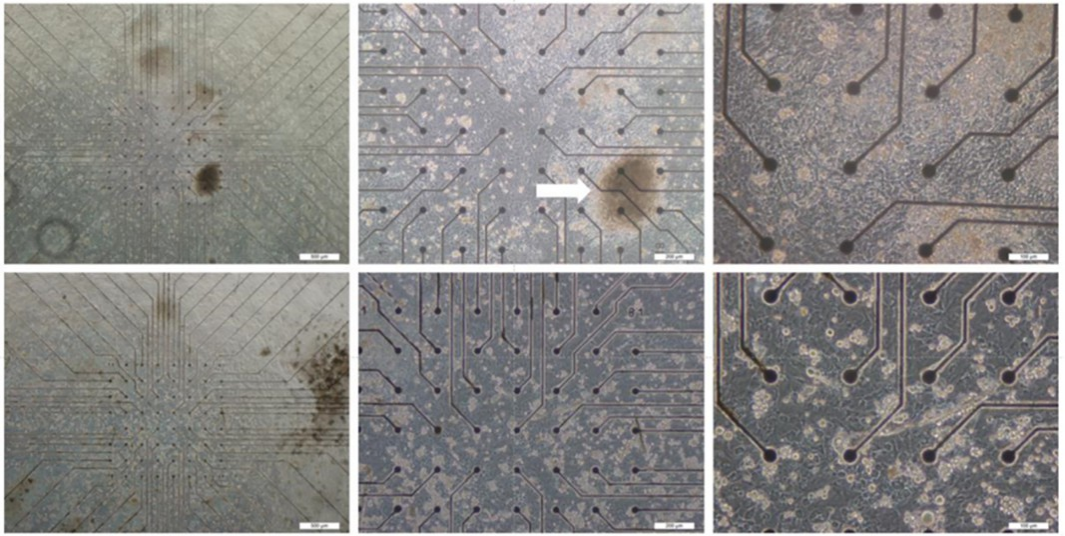
Cardiomyocyte cells cultured on a plate of multi-electrode arrays.

Finally, as mentioned before, in order to compare the results of this method with the plasmonic and electroplasmonic chips, according to the previous method, 18-day-old fetal heart cells of Balb/C mice were used, and the cells were cultured on the 64-electrode plate according to the protocol. The preservation conditions of the cells in the incubator were the same as before. Fig 6 shows an image of heart cells implanted on an electrophysiological plate. As shown in Fig 8, in some parts, the culture was three-dimensional and involved mouse heart microfibers (white arrow).

**Fig 9:**
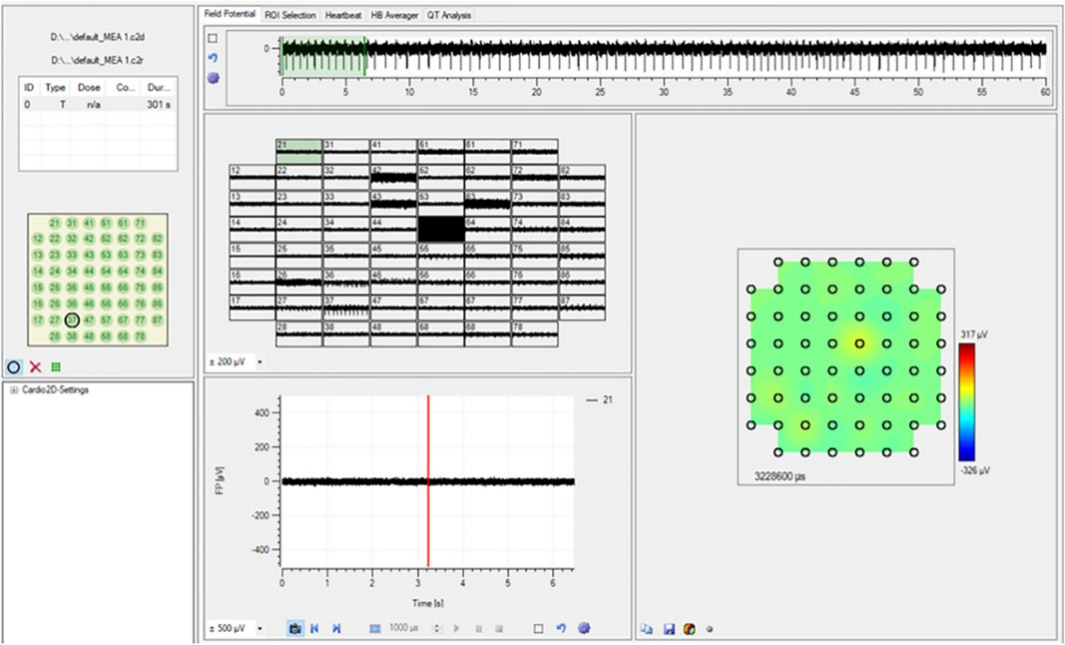
View of offline potential wave analysis software recorded in the channels of a 64-electrode plate.

The extracellular potential was recorded from the cultured cells on the plate. As shown in Fig 9, the plate has electrical waves in channels 27, 37, 55, and 87.

**Fig 10:**
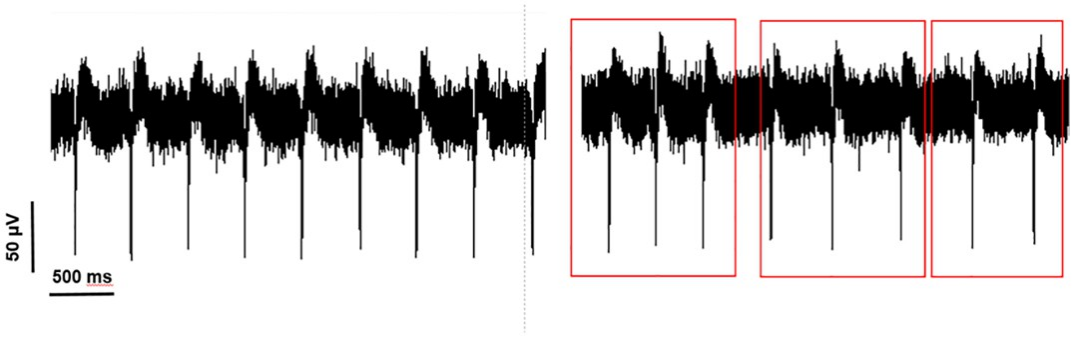
The variable pattern of electrical fluctuations. Red squares show areas with variable patterns.

As a result, the analysis of extracellular potential waves in the target plate shows a variable electrical fluctuation over time. In other words, the heart rhythm is seen in the pattern of electrograms over time (Fig 10).

In detail, for channel 37, the number of beats per minute was 86 bpm and FP_Rise_ = 98 μV and FP_Dur_ = 54 ms. Therefore, by observing the recorded heart signal from the heart cells cultured on the electrophysiology multi-array plate and comparing it with the signals recorded from the heart cells cultured on the plasmonic substrate recorded by our sensor, it can be concluded that our sensor has been able to record the signal with acceptable sensitivity and the results are broadly consistent with the results obtained by electrophysiology method. In addition, our sensor is capable of detecting and recording heart signals in different excitation states with higher signal-to-noise ratio.

**Fig 11:**
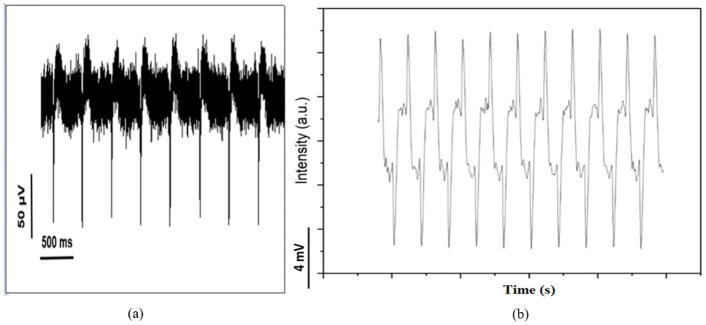
(a). Signal recorded by electrophysiology method. (b). Signal recorded by the plasmonic sensor.

The results obtained by our plasmonic sensor were compared with the results obtained by the electrophysiology method and as it is shown in Fig 11, the results are similar and even the plasmonic sensor has a higher signal-to-noise ratio.

## 4. Conclusion

In this study, a microchip consisting of a red laser with a wavelength of 680 nm and a power of 5 mW, a lens to focus the laser light and a beam splitter, and a photodetector to collect light reflected from the sample surface was first developed. The microchip had many advantages such as small size, lightweight, and portability. Then, one-dimensional plasmonic substrates were prepared as the primary substrate for sensing. After that, the function and accuracy of the generated sensor were evaluated by recording the heart signal of mouse fetal heart cells cultured on the plasmonic substrate in normal states, stimulated by adrenaline and electrical stimulation. The results showed that the designed sensor can record the instantaneous signal of the heart with high accuracy and has enough sensitivity to distinguish between normal and excitation states so that even were able to distinguish between different excitation states and record different signals under different excitation conditions. Finally, the extracellular field potential of cardiomyocyte cells by the electrophysiological method was recorded to confirm our results and it was shown that the recorded electrogram from the heart cell signal by electrophysiology method was very similar to the recorded heart graphs by our sensor. Although all the tests performed in this research were done in vivo and by heart cells extracted from the heart of a mouse fetus, it is hoped that the technique can be developed in the future for real-time detection of the heart rate of a human fetus.

## Supporting information

S1 Data (RAR)
